# Measures of quality of life in children with cochlear implant: systematic review

**DOI:** 10.5935/1808-8694.20130066

**Published:** 2015-10-04

**Authors:** Marina Morettin, Maria Jaquelini Dias dos Santos, Marcela Rosolen Stefanini, Fernanda de Lourdes Antonio, Maria Cecília Bevilacqua, Maria Regina Alves Cardoso

**Affiliations:** a Doctor in Sciences - School of Public Health of the University of São Paulo; Speech and Hearing Therapist, Specialist in Laboratory - School of Dentistry of Bauru - USP.; b Master in Sciences School of Dentistry of Bauru - University of São Paulo (FOB-USP); Speech and Hearing Therapist.; c Specialist in Clinical and Educational Audiology - Craniofacial Anomalies Hospital (HRAC-USP); Speech and Hearing Therapist - Hearing Impaired Patients’ Association, Parents, Friends and Users of Cochlear Implants (ADAP - Bauru).; d Specialist in Clinical and Educational Audiology - Craniofacial Anomalies Hospital (HRAC-USP); Speech and Hearing Therapist - HRAC-USP.; e Full Professor - Department of Speech and Hearing Therapy - Dentistry School of Bauru - University of São Paulo (FOB-USP) and Coordinator of the CPA-HRAC-USP.; f PhD in Public Health; Professor - Department of Epidemiology - School of Public Health of the University of São Paulo - FSP/USP. Department of Epidemiology - School of Public Health - University of São Paulo (USP). Audiological Research Center - Hospital of Craniofacial Anomalies Rehabilitation – University of São Paulo - Bauru Campus.

**Keywords:** child, cochlear implants, hearing loss, quality of life, rehabilitation of hearing impaired

## Abstract

The use of cochlear implant (CI) in children enables the development of listening and communication skills, allowing the child's progress in school and to be able to obtain, maintain and carry out an occupation. However, the progress after the CI has different results in some children, because many children are able to interact and participate in society, while others develop limited ability to communicate verbally. The need for a better understanding of CI outcomes, besides hearing and language benefits, has spurred the inclusion of quality of life measurements (QOL) to assess the impact of this technology.

**Objective:**

Identify the key aspects of quality of life assessed in children with cochlear implant.

**Method:**

Through a systematic literature review, we considered publications from the period of 2000 to 2011.

**Conclusion:**

We concluded that QOL measurements in children include several concepts and methodologies. When referring to children using CI, results showed the challenges in broadly conceptualizing which quality of life domains are important to the child and how these areas can evolve during development, considering the wide variety of instruments and aspects evaluated.

## INTRODUCTION

Several studies have reported that children with severe and/or profound hearing loss (HL) may substantially benefit from using a cochlear implant (CI), together with proper auditory rehabilitation. These children have greater likelihood of acquiring oral language^1^ and being integrated in regular schools^2^, extending their chances of participating in activities and being part of the world of sounds^3^.

To perform activities and participate in the auditory world means to communicate and, consequently, communication is directly related to socializing, since social interactions occur by means of verbal communication^4^. The social aspect is one of the most important parts of a child's global development; it integrates the meaning of “quality of life”, as well as other issues associated with functionality, physical and mental well-being^4^. Therefore, if the CI provides for hearing and language development and, consequently, the development of communication skills, such progress, because of CI use, would bring quality of life improvements for children with hearing loss.

However, although the CI can usually improve the quality of life (QL) of children, there is but a very limited number of studies in our field investigating such aspects^5^. This is a surprising finding, since hearing interference has been well documented, especially in regards of social performance, self-esteem and acceptance at school^6^; and these issues are even more relevant in children with severe and/or profound hearing loss^7^.

Studies in this field evaluate aspects which are more associated with auditory, language and speech performance, school type, and analyze the cost-effectiveness of the CI treatment^1^, ^3^, ^8^, ^9^. More attention has been given to the measures carried through in image/behavior clinic/laboratory than the collection of information at the level of CI user's functionality or other significant factors associated with their bio-psycho-social development^10^.

Concerning the progresses achieved in the field of Audiology, especially with the pediatric population using CI, healthcare professionals must consider that the factors affecting the results are so numerous, and only one part of them can be investigated by means of tests or other instruments used in clinical routine^11^. Moreover, a detailed investigation concerning other aspects of life is not only relevant for the parents and physicians, but also for setting up healthcare policies^12^, allowing for proper resource assignments to take care of the different social needs, service programs and specific interventions for this population^13^.

Thus, to measure health-related quality of life (HRQL), which is a unique and personal perception of physical, mental and social well-being in diverse situations and activities^9^, it is important to evaluate the multidimensional impact of hearing loss and cochlear implant use in the life of children, complementing the results of the clinical measures^14^.

But, specifically in the pediatric population, to measure the HRQL is not an easy task. Numerous methodological issues permeate this type of evaluation, and to measure the state of health of a child requires choices concerning which health aspects are relevant, which preferences are of interest (child, parents, professors, doctors, etc.), the values that must be used, and an entire series of other contextual and psychometric issues that must be tended to^15^.

The challenge is in putting it within a comprehensive concept which HRQL aspects are important for the child, and how such aspects may progress during his/ her development are determining factors in this type of assessment. For example, HRQL domains for a 5 year girl who is starting school can be different from those for an 18 year old who is just starting to drive. This fact directly reflects the choice of instrument to be used, since it must identify and evaluate all the relevant factors for the population being studied. Moreover, most of the time, HRQL questionnaires for children are frequently filled up by the parents or care-givers and studies have shown a poor correlation between the scores from the parents and the child vis-à-vis mental and social aspects, and a better correlation concerning physical domains^16^. Thus, the interpretation of the HRQL results must take into consideration the questionnaire's respondent and, when possible, the evaluation of the parents and that of the child must be done together^17^.

Having all these issues associated with the measure of quality of life in children and trying to guide the bibliography survey with high scientific evidence, we carried through a systematic revision of the literature in order to pinpoint quality of life of children with cochlear implants, and find out which are the main aspects assessed in this population and factors associated with quality-of-life measuring.

## METHOD

As an essential principle of evidence-based studies, the investigated issue in this study was: “Which are the main quality of life aspects assessed in children using CI and the factors related with its results?”.

**Chart 1:**
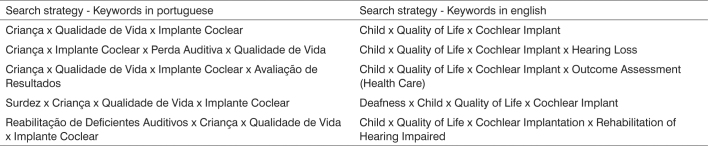
Search strategies in the data bases.

The search strategy used in the bibliographical revision was oriented by the combination of seven keywords indexed in the DecS (health keywords) in Portuguese and English, employing the keywords in groups with at least two keywords (Chart 1).

The chosen scientific databases for the search were: *LILACS, MedLine, SciELO, Cochrane Library, PubMed, Embase, Institute for Scientific Information (ISI)* and *Science Direct.* For the purposes of this study, we considered the publications produced during the period from january of 2000 through september of 2011, and the last manual search was carried out in electronic databases in september of 2011.

The choice of papers followed inclusion criteria based on confining the subject matters to the objectives of this paper. The adopted criteria were:

• Participants - Children with cochlear implants
• Intervention - Cochlear implant
• Measured outcomes - Quality of life by means of questionnaires
• Time - Published in last the 11 years (2000-2011)
• Language: Papers written in Portuguese, English and Spanish
• Types of studies - Papers published in indexed journals with evidence levels 1a, 1b, 2a, 2b, 3a, 3b and 4, in accordance with the criteria proposed by the American Speech Language Hearing Association (ASHA)^18^ (Table 1).

**Table 1:** Levels of scientific evidence according to criteria proposed by the ASHA^18^.

Levels of evidence	Type of study
1a	Systematic review or high-quality metanalysis of randomized controlled trials
1b	High-quality randomized controlled trials
2a	Systematic review or high-quality metanalysis of non-randomized controlled trials
2b	High-quality non-randomized controlled trials
3a	Systematic review of cohort studies
3b	Individual cohort studies or low-quality randomized controlled trials
4	Studies from clinical outcomes
5a	Systematic review of a case-control study
5b	Individual case-control study
6	Series of cases
7	Specialists’ opinion without overt critical assessment

We took off those studies carried out with special groups of children with cochlear implant and other disorders, such as cerebral paralysis, auditory neuropathy, syndromes, auditory nerve hypoplasia, internal component re-implant, bilateral implant and other complications.

The selection of the studies was made in three stages and guided by the above-established criteria. Initially, four revisers analyzed all the studies identified by the combinations of the keywords in all the databases proposed, by analyzing the study title, selecting the papers which gathered the pre-established eligibility criteria (1^st^ stage). Following that, we checked to see if the abstracts had information available on the use of some quality-of-life assessment instrument in children (2^nd^ stage). The cases in which the title or the abstract left margins for doubt we studied the entire texts (3^rd^ stage) to later be deemed pertinent to the subject of study and then be reviewed. The main data for each paper retrieved was carefully collected by means of a standardized protocol for the present study.

A total of 2,937 papers were identified in all the databases. In a pre-selection of these citations, based on reading the titles and summaries of all studies found in the electronic search, we took 2,853 studies off, 84 papers were selected and read in their entirety (Flowchart 1).

**Flowchart 1:**
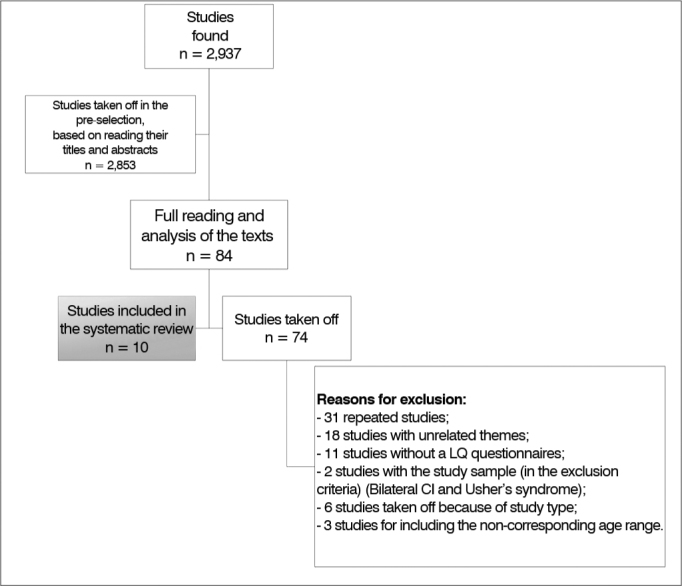
Number of studies identified and selected for inclusion in the systematic review and reasons for exclusion.

At the end, 10 papers met the inclusion criteria^3^, ^7^, ^11^, ^17^, ^19^, ^20^, ^21^, ^22^, ^23^, ^24^. Of these 10 papers included in this revision, 8 were classified as cross-sectional studies^3^, ^11^, ^17^, ^19^, ^20^, ^21^, ^22^, ^23^, ^24^, one was characterized as a high quality non-randomized controlled trial^7^, classified as level 2b according to the ASHA criteria, and one was a systematic revision^17^ (Tables 2 and 3).

**Table 2:** Summary chart of the studies included in the systematic review.

Author and title	Study's objective	Methods/participants	CI information	Questionnaires utilized/aspects assessed	Conclusion
Anmyr L, Olsson M, Larson K, Freijd A. Children with hearing impairment - Living with cochlear implants or hearing aids. International Journal of Pediatric Otorhinolaryngology. 2011;75(6):844-9.	Enhance knowledge on the daily activities of children with CI and ISAD, and their knowledge concerning their hearing and the behavior of others in relation to them.	- Cross-sectional study - Level of evidence ASHA 4 - 74 Children (36 with CI and 38 with ISAD) - Participants’ ages: 6 to 15 years - Control group: Yes - The children answered the questionnaires.	Children who received the CI at 3 years and 10 months; Children who received the ISAD at 5 years and 3 months. No information regarding the CI/ISAD use duration.	Questionnaire based on the CIF-CY. Encompassing the following aspects: 1 - Device use and associated factors 2 - Hearing in different day-to-day situations 3 - Children's perception regarding their hearing and the behaviors of others 4 - Choice of type of communication.	Body Functions: Neck and shoulder pains were significantly more common among children with the ISAD than their CI counterparts; Activities: The children with ISAD use their devices less frequently than the children with CI. More children with CI use sign language than children with ISAD; Participation: More children with ISAD had auditory problems than children with CI while participating in sports and outdoor activities. Children from both groups reported situations in which they would like to hear better, in the classroom for instance, during talks with their families and when they needed to hear at distances; Personal and environmental factors: Many children with CI and ISAD did not find their hearing a problem.

Warner-Czyz AD, Loy B, Tobey EA, Nakonezny P, Roland PS. Health-related quality of life in children and adolescents who use cochlear implants. International Journal of Pediatric Otorhinolaryngology, 2011;75(1):95-105.	Checked the impact of chronological age in the self-assessment results of children with CI on the Health-Related Quality of Life questionnaire (HRQL).	- Cross-sectional study - ASHA 4 level of evidence - 138 children using CI - Age of the participants: 4 to 16 years - Control group: No - Children who answered the questionnaire.	- Received the CI at a mean age of 3.7 years - Mean time using the CI: 5 years.	Using both questionnaires: 1 - KINDLR (Generic HRQL assessment). Covers the following: Physical well-being; emotional well-being; Self-esteem; Family; Friends; School. 2 - One specific for CI, created by the authors. Covering the following aspects: Satisfaction with the CI; Physical well-being; friends; school; self-esteem; social aspects.	Younger children (4-7 years) and using the CI for less time had a more positive HRQL assessment than older children in the KINDLR questionnaire. In the CI-specific questionnaire, the younger group (4-7 years) had a more positive score than the older children. Chronological age differences showed in the CI module in the items associated with friends, school and self-esteem. The issue about the difficulty in hearing the teacher had the opposite effect, in which the younger children reported the worst score. The group of children between 12-16 years was more regular and reliable in their answers.

Sach TH, Barton GR. Interpreting parental proxy reports of (health-related) quality of life for children with unilateral cochlear implants. International Journal of Pediatric Otorhinolaryngology. 2007;71(3): 435-45.	To study which factors are associated with the EuroQol EQ-5D score in children with CI, and to explore the concepts of the parents on health-related quality of life (HRQL) and Quality of Life (QL).	- Cross-sectional study - ASHA 4 evidence level - 216 parents from 222 children with CI (6 parents with two children users of CI) - Mean age of the children: 9.26 years - Control group: No - Parents who answered the questionnaires.	- Age they received the CI: 108 children: < 4 years 114 children: > 4 years - CI time of use: 110 used < 4 years 112 with > 4 years of use.	EQ-5D - Encompasses the following aspects: Mobility; self-care; usual activities; pain; anxiety and depression. Assessment of the health-related quality of life and quality of life, by means of the analogue-visual scale.	They found that the EQ-5D validity construct is variable, although it was capable of discriminating among the children with certain levels of auditory performance, not capable of discriminating among the children who differ in other ways. Moreover, since most of the parents reported that their kid had the same score before and after the implant on a VAS, they inferred that most of the parents rejected the notion that hearing loss was a question of HRQL.

Huber M. Health-related quality of life of Austrian children and adolescents with cochlear implants. International Journal of Pediatric Otorhinolaryngology. 2005;69(8):1089-101.	Aimed at studying the HRQL of school-aged children.	- Cross-sectional study - ASHA 4 level of evidence 4 - 29 children with CI and their parents: 18 children: - Age between 8-12 years; 11 adolescents: ages between 13-16 years - Control group: No - Children and parents answered the questionnaires.	- Mean age in which they received the CI: children: 4.3 years; Adolescents: 7.3 years - Mean time using the CI: children: 6.3 years; Adolescents: 6.9 years.	KINDLR (Assessment of the generic HRQL). Encompasses the following aspects: physical well-being, emotional well-being; self-esteem; family; friends; school.	In comparison with the normal-hearing children, CI children (ages between 8 and 12 years) had below-the-average quality of life, often times perceived by their parents. On the other hand, the adolescents (ages between 13 and 16) reported a normal quality of life.

Schorr EA, Roth FP, Fox NA. Quality of Life for Children With Cochlear Implants: Perceived Benefits and Problems and the Perception of Single Words and Emotional Sounds. Journal of Speech, Language, and Hearing Research. 2009;52(1):141-52.	To study the subjective perceptions of the children regarding their quality of life with the CI, measured by the report of benefits and problems associated with the device and check to see if the CI at an early age could predict their QL perception with their CI.	- Cross-sectional study - ASHA 4 level of evidence - 37 children: 16 boys and 21 girls - Age at the study: 5-14 years (mean of 9 years) - Control group: No - Children who answered the questionnaires.	- Mean age at which they received their CI was 3.2 years - Mean time of use: 5.9 years.	QL questionnaire created for CI children, approaching the following aspects: Frustration; Use of the telephone; Speech understanding; Make new friends; Hear environmental sounds; Academic performance.	The children reported considerable benefits with the CI regarding improvements in their hearing and communication skills and in fields such as social interaction and academic performance. The child has few problems with the CI, especially the conflict with the parents when they do not want to use their CI. Although age at the implant did not predict benefits or problems associated with the CI, the age at which the amplification started predicts the QL score. This implies that early confirmation of the HL and the ISAD use contributes to a positive result in HL.

Loy B, Warner-Czyz AD, Tong L, Tobey EA, Roland PS. The children speak: An examination of the quality of life of pediatric cochlear implant users. Otolaryngology-Head and Neck Surgery. 2010;142(2):247-53.	Checks whether children with CI show, based on their own answers, psychosocial issues similar to those of the hearing counterparts, and if their parents are reliable responders regarding the HRQL of their children.	- Cross-sectional study - ASHA 4 level of evidence - 84 children - 50 children between 8-11 years of age (mean of 9.1 years); and 33 children with 12-16 years of age (mean of 13.7 years) - Control group: Yes (normal hearing children) - Children and parents who answered the questionnaires.	- CI group mean age 8-11 years: 3.37 years, and use duration of 5.71 years - 12-16 years CI group mean age: 5.83 years and use duration of 7.87 years.	KINDLR (Generic HRQL assessment). Covers the aspects: Physical well-being; Emotional well-being; Self-esteem; Family; Friends; School.	In general the QL assessment of the children and adolescents did not differ between the children with CI and normal-hearing children. However, CI children from the 8-11 years group had a more positive score with their families than their normal-hearing counterparts. The 8-11 year-old children had a more positive Q score than the 12-16-year-old group. CI children and parents agreed in the general QL, but the parents of children with CI of 12-16 years overestimated the success of the adolescents at school vis-à-vis the child's self-assessment. The general QL showed a significantly inverse association between the age at implant and a significantly positive correlation with CI use duration in the 12-16 years of age group.

Warner-Czyz AD et al. Parent versus child assessment of quality of life in children using cochlear implants. International Journal of Pediatric Otorhinolaryngology. 2009;73(10):1423-9.	To explore the multidimensional HRQL aspects in 50 CI users between 4 and 7 years of age, by their self-assessment and parents’ assessment.	- High-quality non-randomized controlled trials - ASHA 2b level of evidence - 50 children with CI and their parents. - Mean age at the study: 5 years - Control group: yes (normal hearing children) - Parents and children answered the questionnaires.	- Mean age at which they received the CI: 2.52 years - Mean time of CI use: 3.27 years.	KINDLR (Generic HRQL assessment). Covers the following aspects: Physical well-being; Emotional well-being; Self-esteem; Family; Friends; School.	The children had a significantly more positive QL score than their parents. The total QL had an inversely significant association with the duration of CI use and their chronological age during their assessment. There was no significant correlation between total QL and the age at HL identification or age at surgery. The QL assessment did not differ between children with CI and those with normal hearing.

Huttunen K et al. Parents’ views on the quality of life of their children 2-3 years after cochlear implantation. International Journal of Pediatric Otorhinolaryngology. 2009;73(12):1786-94.	The present study aims at exploring the quality of life of Finish children and families after the CI surgery using a validated questionnaire.	- Cross-sectional study - ASHA 4 level of evidence - 36 children with CI - Mean age at the study: 5 years - Control group: No - Parents who answered the questionnaires.	- Mean age at CI surgery: 3 years and 5 months - 44% used the CI for 2 years and 56% for 3 years.	“Children with cochlear implants: parental perspectives”. Encompasses the following aspects: Communication; General functionality; Self-sufficiency; Well-being and happiness; Social relations; Education.	The CI improved the QL of the children and parents. The parents reported being very much pleased with the quality of life of their children after 2 to 3 years of CI use. The parents’ expectations were better in the following aspects: social relations, communication, general functionality with the CI and child's self-confidence. Speech-intelligibility results were associated with a better development of communication and school performance.

Incesulu A, Vural M, Erkam U. Children With Cochlear Implants: Parental Perspective. Otology & Neurotology. 2003;24(4):605-11.	To assess parents expectations and the progress of child according to the parents.	- Cross-sectional study - ASHA 4 level of evidence - 28 children: 19 boys and 9 girls - Age between 2 and 13 years (CI group mean of 5.07 years) - Control group: No - The parents answered the questionnaires.	- Mean age at which they received the CI: no information - CI time of use between 12 and 30 months (mean 19.5 months).	“Children with cochlear implants: parental perspectives”. Encompasses the following aspects: Communication; General functionality; Self-sufficiency; Well-being and happiness; Social relations; Education.	For the parents, to decide on the cochlear implant surgery is one of the most stressful steps in the process. Although speech and language development had been their main concern, the parents reported noteworthy improvements in their child's communication skills, social relations, and self-confidence. All the families were concerned with device failure.

**Table 3:** Description and systematic review included in the study.

Authors/title/journal	- Lin FR, Niparko JK
	- Measuring health-related quality of life after pediatric cochlear implantation.
	- International Journal of Pediatric Otorhinolaryngology. 2006;70(10):1695-706

Type of study	Systematic review or metanalysis.

Age range	< 18 years.

Investigation question	“How has the HRQL being measured in children with cochlear implant?”

Inclusion criteria	- Original paper;
	- Individuals with ages < 18 years;
	- Children with CI;
	- Studies with quality of life measures, or the functional status or health status;
	- In English.

Results	We found 10 cross-sectional studies, with a minimum age at CI surgery of 2 years. The following quality of life results were discussed in the 10 studies: physiological and psychological well-being; self-confidence, family, friends; general functionality; Communication.
Comments	The results revealed a diversity of literature on QL and CI in children, each one using heterogeneous populations and different QL instruments. Numerous conclusions based on the quality review of the data were found, and they are informative for future investigations.

QV: Quality of life; IC: Cochlear implant.

A systematic review is described on Table 2 with the authors’ names, the year of publication, the journal chosen for publication, type of study, the age group included in the papers examined by the authors, the search string, the inclusion criteria for selecting the studies and the results found.

This set of papers was submitted to data evaluation, and the relevant information from each paper (number of participating subjects, age upon CI, duration of use, mean age of the subjects, object of the study, questionnaires used and conclusion), as well as classification vis-à-vis the degree of recommendation, gathered in tables to facilitate consultation and access during the presentation and result discussion (Table 3).

## RESULTS AND DISCUSSION

The cochlear implant (CI) surgery impact on the children and adolescents with severe and/or profound hearing loss extends to beyond the improvement in hearing and language skills, and in speech production and perception. This impact also involves other aspects of the child's daily life, such as physical, psychological and social well-being^19^.

Considering the interest to investigate which were the main quality-of-life aspects described in the literature and to check which factors were associated with this measure in children and youngsters using CI, this study involved a systematic survey of the literature in this field.

The results showed a difference among the studies investigated, considering the age upon evaluation, age of surgery, CI use duration, and the instruments used to assess quality of life. These results were also found in the systematic review carried out by Lin & Niparko^17^.

From the qualitative analysis of the studies, it was possible to notice that the main aspects of quality of life raised in the studies selected for this systematic review were: physical well-being; emotional well-being; self-esteem; family; friends; school; satisfaction with the CI; social aspects; mobility; self-care; pain; telephone use; speech understanding; hearing environmental sounds; communication; self-sufficiency; use of the devices; attitudes of the others and self-confidence.

Thus, both aspects of health-related quality of life (physical, psychological and social well-being)^20^ and specific aspects of this population of CI users (family, friends, school, satisfaction with the CI, telephone use, speech understanding, listening to environmental sounds and use of the devices) were investigated.

Although the generic instruments of health-related quality-of-life evaluation are much too general vis-à-vis the investigated aspects, which cannot enable the investigation of issues of particular interest for a given condition (for instance, telephone use), some studies currently show that these bear enough sensitivity, given the ample impact that the hearing loss has on the life of a child^³^. Another advantage of this type of instrument is the ability of being able to compare the multidimensional aspects that make it, in different groups of children^20^.

Moreover, currently few specific and standardized HRQL assessment tools are available for the pediatric population with hearing loss. It was only in December of 2011 that a tool intended for the assessment of quality of life in children with hearing loss was published, called *“Hearing Environments and Reflections on Quality of Life* (HEAR-QL)”^25^. This questionnaire was not translated into Portuguese until the final analysis of this study.

Thus, we recommend that these two types of assessment should be used in order to perform a HRQL assessment in children with hearing loss, as complementary to the clinical results. The two instruments are needed to completely understand the CI impact instead of compartmentalizing this intervention into an auditory phenomenon only^20^.

In relation to the analysis of the quality-of-life measure-related factors in children and youngsters with CI, one of the evaluated aspects was the child's age upon surgery. The qualitative analysis of the studies which ran this analysis made it possible to consider that children who were submitted to surgery in earlier ages make a more positive analysis of their quality of life.

Although each study evaluated children at different ages, research in this field show that children implanted earlier reach a better auditory perception, better, incidental language acquisition and better speech inteligibility^1^, ^8^. The early development of these skills can improve the children's communication with their parents and at school, thus bringing about better social performances, reflected on quality of life assessments.

As to the duration of use, of the three studies that ran this analysis, two found a positive correlation between the total HRQL score and the duration of the CI use, and those children using it longer had a more positive assessment of their HRQL. This aspect has also has been relevant in the results obtained from children^26^. Children using it longer and more effective may have a better speech perception and intelligibility performances; and just like the age upon implantation, the more effective communication may bring about benefits for other aspects of life.

Thirty children using CI for a period of 10 to 14 years were assessed in a prospective and longitudinal study as to their speech perception and intelligibility. The results showed that 87% of the children used the implant effectively, and after 10 years of use, 60% could speak on the telephone, and 77% developed speech intelligibility near that of their normal hearing counterparts^27^.

Some studies^7^, ^20^, ^21^ found a significantly inverse correlation between the child's chronological age and the HRQL evaluation, in which the younger children made a more positive classification of their HRQL than the older children. The groups of children evaluated in these studies had ages varying between 4 and 16 years and, in the three studies, the younger children had been submitted to the CI surgery earlier than the older children, and these findings can be justified vis-à-vis quality of life.

This early identification and prevention of the hearing loss may have provided for a faster and more complete acceptance, recover of hearing and, consequently, of the CI in the lives of the younger children. That is, the CI use within the children's day-to-day activities enables them to embody the device as part of themselves, instead of being something that distinguished them from their normal-hearing colleagues^7^.

Both for children and their parents, the speech perception results were correlated with quality of life, and these findings may indicate that their perceptions regarding the well-being of the CI users are influenced by factors that go beyond hearing and communication capacity^28^. Moreover, today, advances in the CI hardware, *software* and speech processing technology have had a direct impact on the performance and success associated with speech understanding, and such factors should be always considered, since they may in such a way impact the QL results.

As to the differences in quality-of-life evaluations among children using the CI, their parents and children with hearing, the data did not allow for conclusions in relation to these comparisons.

In some fields, such as auditory rehabilitation - where the problems are of complex and intervention cannot be done in definitive groups (control group *versus* case group), it is possible to include in the systematic review those studies with limited methodological characteristics, at least for the methodological standards adopted by high scientific evidence studies^29^. Consequently, these studies could be susceptible to a restricted analysis, but they should not be discarded.

We must consider the different ages at which the surgery was carried out, and the duration of CI use in each study must be considered a limitation, given the well-established association between the development of hearing and language skills and these variables and, therefore, the heterogeneity of these factors may result in a population with broad results vis-à-vis language skills^17^.

## FINAL REMARKS

Further studies must be done, using HRQL assessment tools which enable result comparison among clinics and countries, and which may lead to a better understanding of the criteria used to select candidates for the surgery, the needs for rehabilitating children with CI, besides enabling access to the clinics, allowing the children with CI to develop their true potential in all aspects of their lives.
